# Supplementary material for: Promote Community Engagement in Participatory Research for Improving Breast Cancer Prevention: The P.I.N.K. Study Framework
Source: Cancers (Basel). 2022 Nov 25;14(23):5801. doi: 10.3390/cancers14235801 (PMC9736257; doi:10.3390/cancers14235801)
Supplement: Supplementary file 1 [file cancers-14-05801-s001.zip › cancers-1999807-supplementary.pdf]

Appendix B

Table S1 - Distribution of the characteristics included in the profile 1: Fuchsia family and focus on each combination of communities within the Fuchsia family

	**Combinations of communities within the Fuchsia family**								
**Profile 1 - non modifiable factors:**	Fuchsia	Fuchsia	Fuchsia	Fuchsia	Fuchsia	**chi-square test**	**Fuchsia combinations other than FYF**	**chi-square test**	**Overall Fuchsia family**
**Profile 2 - personal history**	Yellow	Yellow	Yellow	Yellow	Yellow				
**Profile 3 - lifestyle habits**	Fuchsia	Yellow	Green	Blu	Azure				
**Combination**	**FYF**	**FYY**	**FYG**	**FYB**	**FYA**				
**Number of women**	193	360	136	342	268		1,106		1,299
**BC prevalence**	4.7%	1.9%	2.2%	2.3%	1.9%	p>0.05	2.1%	p=0.033	2.5%
[95% CI]	*[2.1-8.7%]*	*[0.8 -4.0%]*	*[0.5 -6.3%]*	*[1.0 -4.6%]*	*[0.6-4.3%]*		*[1.4 -3.1%]*		[1.7-3.5%]
**Years of age (average±SD)**	68.5 (±6.5)	64.8 (±7.3)	62.8 (±3.6)	63.2 (±7.0)	64.0 (±6.9)		63.8 (±6.8)		64.5 (±6.9)
focus on age classes			50-54 **(b)**						
**Breast density**						p>0.05		p>0.05	
BI-RADS A (less dense)	29.5%	29.7%	24.3%	26.3%	31.7%		28.5%		28.6%
BI-RADS B	44.6%	45.3%	44.9%	46.5%	46.6%		45.9%		45.7%
BI-RADS C	23.8%	23.3%	27.2%	25.7%	20.9%		24.0%		23.9%
BI-RADS D	2.1%	1.7%	3.7%	1.5%	0.7%		1.6%		1.7%
**Family history**						p>0.05		p>0.05	
no first line relatives' BC	80.8%	79.2%	76.5%	79.5%b	76.1%		78.2%		78.6%
first line female relative's BC	19.2%	20.8%	23.5%	20.5%b	23.9%		21.8%		21.4%
first line male relative's BC	0.0%	0.0%	0.0%	0.0%a	0.0%		0.0%		0.0%
first line male and female relatives' BC	0.0%	0.0%	0.0%	0.0%b	0.0%		0.0%		0.0%
**Age of menarche (Avg. years±SD)**	12.5 (±1.3)	12.4 (±1.4)	12.3 (±1.3)	12.3 (±1.4)	12.3 (±1.4)		12.4 (±1.4)		12.4 (±1.4)
**Reproductive period length**	38.5 (±3.8)	39.1 (±3.6)	38.8 (±4.1)	38.7 (±4.0)	38.9 (±4.3)	p>0.05	38.9 (±3.9)	p>0.05	38.8 (±3.9)
**Menopause status length (Avg. years±SD)**	17.5 (±7.8)	13.3 (±7.8)	11.7 (±5.0)	12.1 (±7.3)	12.7 (±7.8)		12.6 (±7.4)		13.3 (±7.7)
focus on lenth classes	≤5(-);>5(+)					p>0.05	≤5(+);>5(-)	p=0.000	

Association between each risk factor and each Fuchsia combination (chi-square test p< 0.05): *negative (adj. residual <-2); **positive (adj. residual >2);

Association between BC cases and each risk factor by Fuchsia combinations (chi-square test p<0.05): (a) negative (adj. res. <-2); (b) positive (adj. res. >2)
Association between each risk factor in the comparison of the FYF and the group of the other Fuchsia combinations (chi-square test p< 0.05): (-) negative (adj. residual <-2); (+) positive /adj. residual >2);
Association between BC cases and each risk factor in the comparison of the FYF and the group of the other Fuchsia combinations (chi-square test p<0.05): (c) positive (adj. residual >2)

Table S2 - Distribution of the characteristics included in the profile 2: Fuchsia family and focus on each combination of communities within the Fuchsia family

	**Combinations of communities within the Fuchsia family**								
**Combination**	**FYF**	**FYY**	**FYG**	**FYB**	**FYA**	**chi-square test**	**Fuchsia combinations other than FYF**	**chi-square test**	**Overall Fuchsia family**
**Number of women**	193	360	136	342	268		1,106		1,299
**BC prevalence**	4.7%	1.9%	2.2%	2.3%	1.9%	p>0.05	2.1%	p=0.033	2.5%
[95% CI]	*[2.1-8.7%]*	*[0.8 -4.0%]*	*[0.5 -6.3%]*	*[1.0 -4.6%]*	*[0.6-4.3%]*		*[1.4 -3.1%]*		[1.7-3.5%]
**Qualification**						p<0.001		p=0.009	
primary or secondary education	44.0%**(+)	33.9%**(b)**	29.4%	26.6%*	40.7%**		32.7%*(-)		34.4%
high school graduation	30.6%	35.8%**(a)**	36.0%	35.1%	36.6%		35.8%		35.0%
university or post graduate degree	25.4%	30.3%	34.6%	38.3%**	22.8%*		31.5%		30.6%
**Occupation**						p<0.001		p<0.001	
unemployed/housewife	74.1%**(+)	62.8%	55.9%	53.5%*	53.4%*		56.8%(-)		59.3%
employed	20.7%*(-)	32.8%	41.2%	40.9%**	42.9%**		38.8%(+)		36.1%
retired	5.2%	4.4%	2.9%	5.6% **(b)**	3.7%		4.4%		4.5%
employed/retired who worked night shifts	0.0%	0.0%	0.0%	0.0%	0.0%		0.0%		0.0%
**Current/past Co-morbid.** (yes)						p>0.05		p>0.05	
1 co-morbidity	20.7%	21.9%	28.7%	28.9%	25.0%		25.7%		24.9%
at least 2 co-morbidities groups:	71.0%	70.3%	65.4%	63.7%	69.4%		67.5%		68.0%
Cardiometabolic Diseases	71.0%	70.3%	69.9%	67.0%	75.0%	p>0.05	70.3%	p>0.05	70.4%
Endocrine Diseases	52.8%	52.5%	56.6%	49.7%	51.9%	p>0.05	52.0%	p>0.05	52.1%
Intestinal Diseases	16.1%	12.5%	8.8%	12.9%	13.8%	p>0.05	12.5%	p>0.05	13.0%
Neuropsychiatric Diseases	42.5%	46.9%	41.2%	43.3%	45.9%	p>0.05	44.8%	p>0.05	44.5%
Autoimmune disease	36.3%(+)	27.5%	27.9%	29.8%	30.6%	p>0.05	29.0%(-)	p=0.043	30.1%
**Chest radiation therapy** (yes)	13.5%	15.6%	5.9%*	12.3%	17.2%	p=0.023	13.7%	p>0.05	13.7%
**Pregnancy experience**(at least one)	90.2%	91.4%	87.5%	87.4%	86.9%	p>0.05	88.6%	p>0.05	88.8%
**Breast feeding experience** (yes)	74.6%	74.7%	73.5%	73.7%	69.4%	p>0.05	73.0%	p>0.05	73.2%

**BMI**						p=0.011		p>0.05	
underweight (<18.5)	0.0%	0.3%	0.0%	1.2%****(b)**	0.0%		0.5%**(b)**		0.4%
normal weight (18.5 - 24.9)	53.9%	55.8%	61.0%	64.3%**	47.0%*		57.0%		56.5%
overweight (25 - 29.9)	35.2%	31.1%	25.7%	23.7%*	32.8%		28.6%		29.6%
obese (≥30)	10.9%	12.8%	13.2%	10.8%	20.1%**		14.0%		13.5%
**Weight gain in menopause**						p=0.012		p>0.05	
yes	63.2%	70.0%	64.7%	63.7%	75.4%**		68.7%		67.9%
**Oral contraceptive use**						p=0.001		p<0.001	
never or less than 1 year	74.1%**(+)	59.2%	50.0%*	56.7%	56.7%		56.7%(-)		59.3%
1-5 years	16.1%*(-)	22.5%	29.4%**	22.8%	22.4%		23.4%(+)		22.3%
more than 5 years	9.8%*(-)	18.3%	20.6%	20.5%	20.9%		19.9%(+)		18.4%
**Hormone therapy for ovarian stimulation use**						p>0.05		p>0.05	
at least 1 cycle of stimulation	1.0%	2.2%	3.7%	3.8%	3.7%		3.3%		2.9%
**Hormone replacement therapy use (HRT)**						p>0.05		p>0.05	
more than 6 month	23.8%	22.8%	17.6%	27.8%	23.9%		24.0%		23.9%

Association between each risk factor and each Fuchsia combination (chi-square test p< 0.05): *negative (adj. residual <-2); **positive (adj. residual >2);
Association between BC cases and each risk factor by Fuchsia combinations (chi-square test p<0.05): (a) negative (adj. res. <-2); (b) positive (adj. res. >2)
Association between each risk factor in the comparison of the FYF and the group of the other Fuchsia combinations (chi-square test p< 0.05): (-) negative (adj. residual <-2); (+) positive /adj. residual >2);
Association between BC cases and each risk factor in the comparison of the FYF and the group of the other Fuchsia combinations (chi-square test p<0.05): (c) positive (adj. residual >2)

Table S3 - Distribution of the characteristics included in the profile 3: Fuchsia family and focus on each combination of communities within the Fuchsia family

	**Combinations of communities within the Fuchsia family**					**chi-square test**	**Fuchsia combinations other than FYF**	**chi-square test**	**Overall Fuchsia family**
**Combination**	**FYF**	**FYY**	**FYG**	**FYB**	**FYA**				
**Number of women**	193	360	136	342	268		1,106		1,299
**BC prevalence** [95% CI]	4.7% [2.1-8.7%]	1.9% [0.8 - 4.0%]	2.2% [0.5 - 6.3%]	2.3% [1.0 - 4.6%]	1.9% [0.6-4.3%]	p>0.05	2.1% [1.4 - 3.1%]	p=0.033	2.5% [95%CI: 1.7-3.5%]
**Current and past smoking habit**						p<0.001		p<0.001	
never-smokers	68.4%**(+)	38.6%	30.1%*	37.1%*	43.3%		38.2%(-)		42.7%
1-5 cigarettes/day	15.0%*(-)	40.0%**	17.6%*	31.3%	23.5%		30.6%(+)		28.3%
6-10 cigarettes/day	8.8%*(-)	9.4%*	41.9%**	9.6%*	18.7%**		15.7%(+)		14.7%
more than 10 cigarettes/day	7.8%*(-)	11.9%	10.3%	21.9%**	14.6%		15.5%(+)		14.3%
**Alcohol drinking habit:**						p<0.001		p<0.001	
no alcohol drinker	46.1%**(+)	19.7%*	17.6%	16.1%*	27.6%		30.3%(-)		24.1%
one time per week or less	34.7%*(-)	43.9%	56.6%**	56.4%**	46.3%		49.9%(+)		47.7%
more times per week	19.2%*(-)	36.4%**	25.7%	27.5%	24.3%		29.4%(+)		27.9%
binge-drinking at least one time per week	0.0%	0.0%	0.0%	0.0%	1.9%**		0.5%		0.4%
**WCRF recommendations compliance:**									
1. Stay physically active every day						p<0.001		p>0.05	
low	36.3%**(b)(c)**	30.8%*	27.9%*	22.5%*	62.3%**		35.5%		35.6%
medium	42.0%	51.4%**	41.9%	34.2%*	32.1%*		40.2%		40.5%
high/very high	21.8%	17.8%*	30.1%	43.3%**	5.6%*		24.2%		23.9%
2. Limit energy-dense foods and avoid sugary drinks						p<0.001		p>0.05	
low	18.1%	15.3%*	16.9%	9.1%*	60.4%**		24.5%		23.6%
medium	29.5%	46.9%**	27.9%	16.1%*	21.6%*		28.9%		29.0%
high/very high	52.3%	37.8%*	55.1%	74.9%**	17.9%*		46.6%		47.4%

3. Follow a diet mainly based on plant foods, cereals, fruit and legumes						p<0.001		p<0.001	
low	4.7%*(-)	7.2%*	11.8%	2.6%*	41.8%**		14.7%(+)		13.2%
medium	49.7%**	60.0%**	39.7%	25.4%*	35.4%*		40.9%		42.2%
high/very high	45.6%(+)	32.8%*	48.5%	71.9%**	22.8%		44.4%(-)		44.6%
4. Limit the consumption of red meats and cured meats						p<0.001		p<0.001	
low	5.2%*(-)	8.6%*	13.2%	5.6%***(b)**	42.9%**		16.5%(+)		14.9%
medium	42.0%	60.3%**	32.4%	21.9%*	37.7%		39.5%		39.9%
high/very high	52.8%**(+)	31.1%*	54.4%**	72.5%**	19.4%*		43.9%(-)		45.3%
5. Limit the consumption of salt						p<0.001		p=0.001	
low	6.7%*(-)	6.9%*	9.6%	5.8%*	44.8%**		16.1%(+)		14.7%
medium	35.8%	52.5%**	39.7%	20.8%*	33.2%		36.4%		36.3%
high/very high	57.5%**(+)	40.6%*	50.7%	73.4%**	22.0%*		47.5%(-)		49.0%
6. Follow a varied diet						p<0.001		p=0.027	
low	2.1%*(-)	0.6%*	5.9%	1.5%*	24.3%**		7.2%(+)		6.5%
medium	45.6%	57.2%**	41.2%	21.9%*	52.2%**		43.1%		43.5%
high/very high	52.3%	42.2%*	52.9%	76.6%**	23.5%*		49.6%		50.0%

Association between each risk factor and each Fuchsia combination (chi-square test p< 0.05): *negative (adj. residual <-2); **positive (adj. residual >2);
Association between BC cases and each risk factor by Fuchsia combinations (chi-square test p<0.05): (a) negative (adj. res. <-2); (b) positive (adj. res. >2)
Association between each risk factor in the comparison of the FYF and the group of the other Fuchsia combinations (chi-square test p< 0.05): (-) negative (adj. residual <-2); (+) positive /adj. residual >2);
Association between BC cases and each risk factor in the comparison of the FYF and the group of the other Fuchsia combinations (chi-square test p<0.05): (c) positive (adj. residual >2)
